# Trop-2 is a novel target for solid cancer therapy with sacituzumab govitecan (IMMU-132), an antibody-drug conjugate (ADC)*

**DOI:** 10.18632/oncotarget.4318

**Published:** 2015-06-18

**Authors:** David M. Goldenberg, Thomas M. Cardillo, Serengulam V. Govindan, Edmund A. Rossi, Robert M. Sharkey

**Affiliations:** ^1^ Immunomedics, Inc., Morris Plains, NJ, USA

**Keywords:** antibody-drug conjugate, Trop-2, SN-38, solid cancers, triple-negative breast cancer

## Abstract

Trop-2 is a novel target for ADC therapy because of its high expression by many solid cancers. The rational development of IMMU-132 represents a paradigm shift as an ADC that binds a well-known moderately-cytotoxic drug, SN-38, to the anti-Trop-2 antibody. *In vitro* and *in vivo* studies show enhanced efficacy, while there is a gradual release of SN-38 that contributes to the overall effect. IMMU-132 is most efficacious at a high drug:antibody ratio (DAR) of 7.6:1, which does not affect binding and pharmacokinetics. It targets up to 136-fold more SN-38 to a human cancer xenograft than irinotecan, SN-38′s prodrug. IMMU-132 delivers SN-38 in its most active, non-glucuronidated form, which may explain the lower frequency of severe diarrhea than with irinotecan. Thus, this ADC, carrying a moderately-toxic drug targeting Trop-2 represents a novel cancer therapeutic that is showing promising activity in patients with several metastatic cancer types, including triple-negative breast cancer, non-small-cell and small-cell lung cancers.

## INTRODUCTION

A novel target in multiple solid cancers for an ADC is trophoblast cell-surface antigen, or Trop-2, also known as tumor-associated calcium signal transducer (TACSTD2), epithelial glycoprotein-1 (EGP-1), gastrointestinal tumor-associated antigen (GA733-1), and surface marker 1 (M1S1) [1, 2]. The expression, role, and function of Trop-2 have been of interest since about 1990, when our anti-Trop-2 monoclonal antibody was shown to bind to many different cancer types [3, 4]. It is encoded by a single-copy gene (*TACSTD2*) mapped on chromosome 1p32 [5], which hybridizes to a single 1.8-kb mRNA encoding the *GA733-1* gene [6]. The 36 kDa nascent polypeptide, which is post-translationally modified by N-linked glycosylation, forms a type-1 transmembrane protein that is distinct from EpCAM (EGP-2) [3, 7]. First described as a cell-surface glycoprotein of a human trophoblast cell, Trop-2 was believed at that time to be involved in regulating the growth and invasion of cancer cells [8–10]. The *Trop-2/TACSTD2* gene has been cloned [8] and found to encode a transmembrane Ca^++^-signal transducer [1, 11]. Functionally, it is linked to cell migration and anchorage-independent growth, with higher expression in a variety of human epithelial cancers, including breast, lung, gastric, colorectal, pancreatic, prostatic, cervical, head-and-neck, and ovarian carcinomas, compared to normal tissues [2, 7, 12, 13]. The increased expression of Trop-2 is reported to be necessary and sufficient for stimulation of cancer growth [13], while a bi-cistronic cyclin D1-Trop-2 mRNA chimera is an oncogene [14]. Importantly, elevated expression is associated with more aggressive disease and a poor prognosis in several cancer types [12, 14–19], including breast cancer [20, 21]. Increased *Trop-2* mRNA is a strong predictor of poor survival and lymph node metastasis in patients with invasive ductal breast cancers, and Kaplan-Meier survival curves show that breast cancer patients with high *Trop-2* expression have a significantly shorter survival [21]. Using genomic analyses of breast cancers, it was proposed that Trop-2 is a potentially attractive target for triple-negative breast cancer (TNBC) [22], which we reported with RS7 anti-Trop-2 antibody conjugated to a radionuclide [23].

We are assessing the clinical role of a new Trop-2-targeting ADC using the humanized RS7 antibody as a potentially improved treatment for diverse epithelial cancers, including TNBC (http://ClinicalTrials.gov number NCT01631552). This ADC, designated IMMU-132, is important because it represents a significant departure from the current ADC paradigm of using a stably-linked ultratoxic drug by: (i) use of a moderately-toxic drug, SN-38, (ii) conjugation of drug to monoclonal antibody (mAb) at a high ratio (∼8:1) without affecting antibody targeting and pharmacokinetics, (iii) utilization of a pH-sensitive, cleavable linker designed to impart cytotoxic activity to both target and bystander cells via ADC internalization and local release of the free drug at the tumor, (iv) allowing high doses of the ADC over a prolonged times without provoking an immune response, and (v) showing reduced toxicities, especially a lower incidence of severe diarrhea, which is common for topoisomerase inhibitors.

In this article, we report that Trop-2 is an attractive target for an ADC, especially since RS7 internalizes rapidly into target cancer cells [4]. Preclinical results, supported by an ongoing clinical trial, highlight the attributes distinguishing this anti-Trop-2-targeting ADC as a novel agent for the treatment of patients with relapsed/refractory, metastatic solid cancers [24], especially triple-negative breast cancer (TNBC) [25]. We also demonstrate, for the first time, that a moderately-toxic drug can be conjugated to a cancer-targeting antibody and show an improved therapeutic index that is predictive of this ADC's clinical activity.

## RESULTS

### Humanized anti-Trop-2 antibody

The RS7 antibody was developed against a human squamous cell carcinoma of the lung, binding specifically to a∼45 kDa glycoprotein initially denoted EGP-1 [3, 4]. It was later determined to be identical to an antigen defined earlier by Lipinski et al. [9] as Trop-2, which is now the more commonly used designation. The murine anti-Trop-2 mAb, designated RS7-3G11 (or RS7) [4], was humanized to reduce immunogenicity for clinical use. Antigen-binding for Trop-2^+^ cell lines, as well as rapid cell internalization, were preserved in the ADC (e.g., K_D_ is 0.564 ± 0.055 nM and 0.658 ± 0.140 nM, hRS7 IgG and IMMU-132, respectively) [2].

### Structure and properties

**Figure 1:**
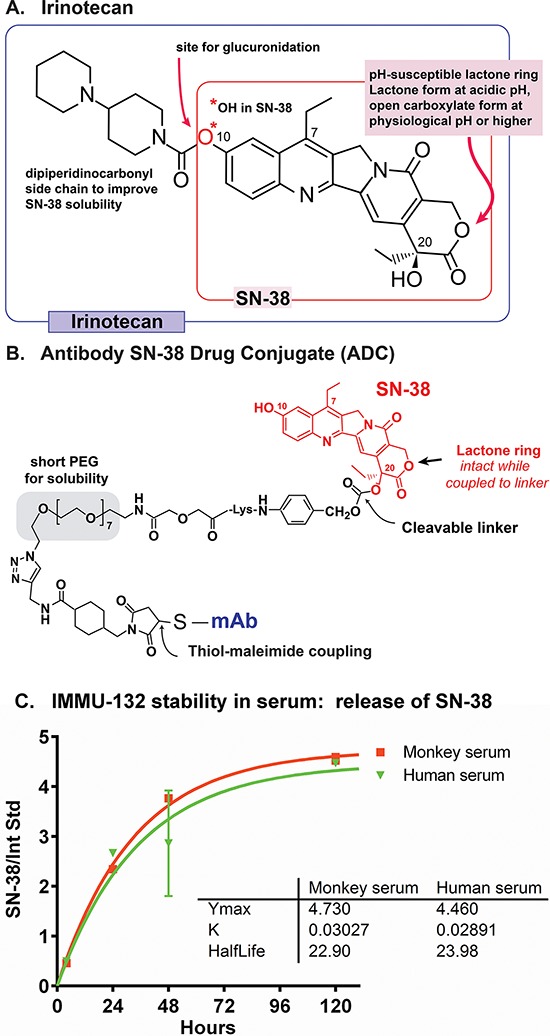
Structures of irinotecan, SN-38, and CL2A-SN-38 **A.** Irinotecan schematic indicates the molecule's 7, 10, & 20 positions. **B.** IMMU-132 ADC shows SN-38 positioning, site of SN-38 cleavage, PEG moiety to enhance solubility, and site coupled to the antibody. **C.** *In vitro* serum stability of IMMU-132 in monkey or human serum.

IMMU-132 utilizes the topoisomerase I inhibitor, SN-38, the water insoluble metabolite of the anticancer camptothecin, irinotecan (7-ethyl-10-[4-(1-piperidino)-1-piperidino]carbonyloxycamptothecin) (Fig 1), which is therapeutically active in colorectal, lung, cervical, and ovarian cancers [26]. An important advantage for selecting SN-38 is that the drug's *in-vivo* pharmacology is well known. Irinotecan must be cleaved by esterases to form SN-38, which is 2–3 orders of magnitude more potent than irinotecan, with activity in the low nanomolar range [27]. At physiological pH, camptothecins exist in an equilibrium comprising the more active lactone form and the less active (10% potency) open carboxylic acid form [28, 29].

The design of the SN-38 derivative used in IMMU-132, CL2A-SN-38 [2, 30, 31], addressed multiple challenges in using this drug in the ADC format, and involved the following features: (i) a short polyethylene glycol (PEG) moiety was placed in the cross-linker to confer aqueous solubility to this highly insoluble drug (Fig 1B); (ii) a maleimide group was incorporated for fast thiol-maleimide conjugation to mildly reduced antibody; (iii) a benzylcarbonate site provided a pH-mediated cleavage site to release the drug from the linker; and (iv) importantly, the crosslinker was attached to SN-38′s 20-hydroxy position, which kept the lactone ring of the drug from opening to the less active carboxylic acid form under physiological conditions [29, 32]. The synthesis of SN-38 derivatives and the conjugation of CL2A-SN-38 to mildly reduced hRS7 IgG has been described previously [2, 30, 31]. The limited reduction procedure breaks only the interchain disulfide bridges between the heavy-heavy and heavy-light chains, but not the intra-domain disulfides, generating 8 site-specific thiols per antibody molecule. It is then conjugated to CL2A-SN-38, purified by diafiltration, and lyophilized for storage. During manufacturing, conditions are adjusted to minimize any loss of SN-38 from IMMU-132, with the final lyophilized product consistently having < 1% free SN-38 when reconstituted. However, when placed in serum and held at 37°C, SN-38 is released from the conjugate with a half-life of ∼1 day (Fig 1C).

This represents a marked departure from the two recently reported ADCs utilizing ultratoxic drugs, where their linkers maintain a relatively high degree of stability in serum [33, 34]. The release of SN-38 appears to be an important feature of IMMU-132, with this type of linker selected based on efficacy studies that tested SN-38 conjugated to a variety of linkers that had different rates of SN-38 release, ranging from ∼10 h release half-life to being highly stable [30, 31]. Optimal therapeutic activity was found with a conjugate having an intermediate release rate in serum of ∼1 day. We subsequently improved the manufacturing process for this type of linker, designated CL2A [2], and then again compared the efficacy of this conjugate to another stably-linked anti-Trop-2 conjugate that was designed to release SN-38 only under lysosomal conditions (i.e., in the presence of cathepsin B and pH 5.0) at a rate similar to that as CL2A-linked SN-38 under identical conditions. In animal models, the anti-Trop-2 conjugate prepared with the CL2A linker yielded better therapeutic responses than when SN-38 was linked stably, indicating that even antibodies that internalized quickly benefitted when SN-38 was allowed to be released in serum with a half-life of ∼1 day [35]. Since clinical studies with radiolabeled antibodies have found the antibodies localize in tumors within a few hours, reaching peak concentrations within 1 day [36], selectively enhanced concentrations of SN-38 are delivered locally in the tumor through internalization of the intact conjugate, extracellular release of the free drug, or both mechanisms in concert.

### Drug-antibody ratio (DAR) determination

**Figure 2:**
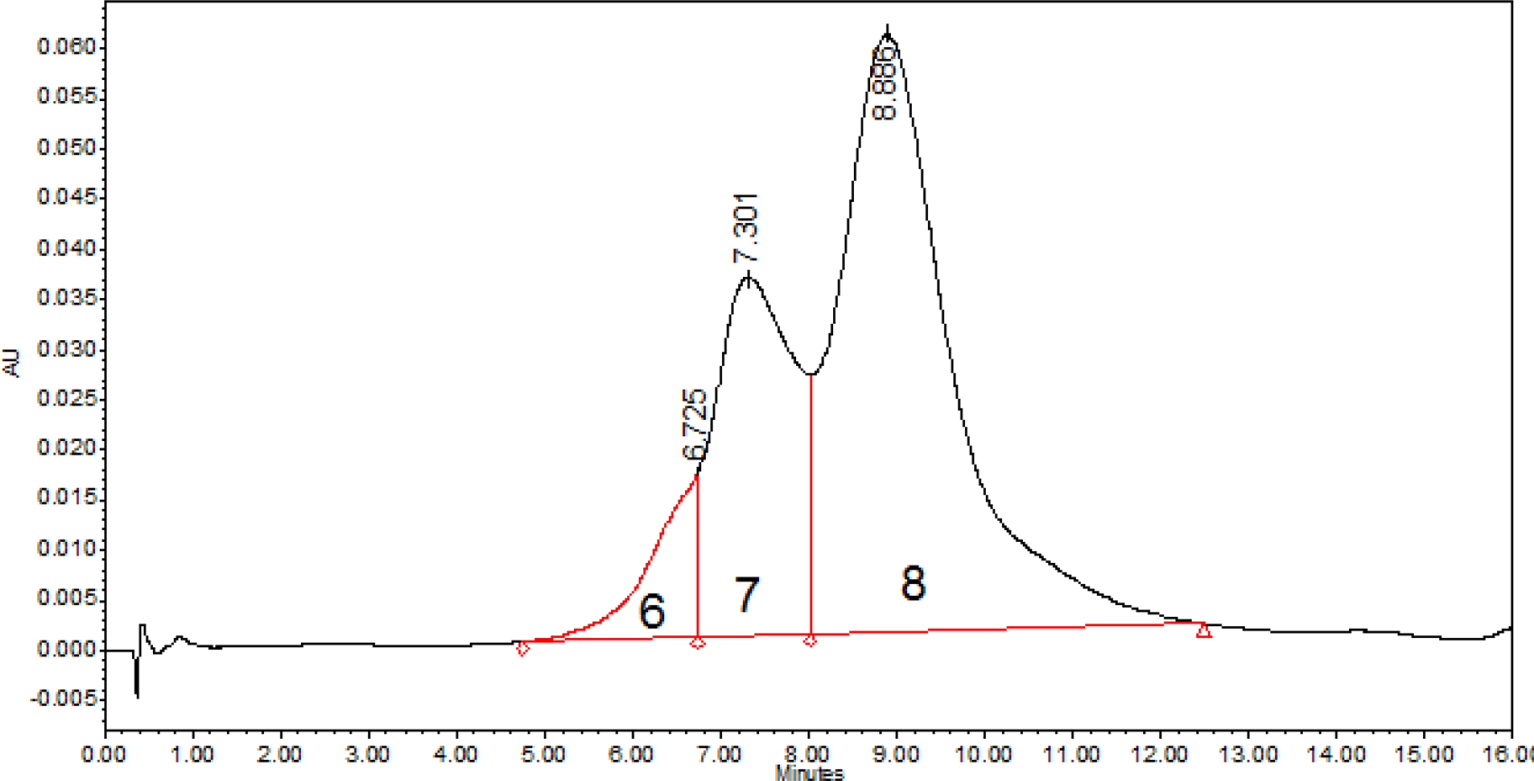
Hydrophobic interaction chromatography of IMMU-132 Representative HIC-HPLC trace of IMMU-132 resolved with a 15-min gradient of 2.25 M to 1.5 M NaCl. Peaks representing species with DARs of 6, 7 and 8 are indicated.

Five clinical lots of IMMU-132 were evaluated by hydrophobic interaction HPLC (HIC-HPLC), which resolved three peaks representing species with DARs of 6, 7 and 8, with the greatest fraction comprising a DAR = 8 (Fig 2). IMMU-132 was produced consistently by this manufacturing process, with an overall DAR (DAR_AVE_) of 7.60 ± 0.03 among five clinical lots (Table 1). HIC-HPLC results were confirmed by liquid chromatography-mass spectrometry (LC-MS) (Table 2). The analysis showed that > 99% of the 8 available sulfhydryl groups were coupled with the CL2A linker, either with or without SN-38. There were no unsubstituted [or *N*-ethylmaleimide (NEM) capped] heavy or light chains detected. Thus, the DAR is slightly less than 8 because a small amount of SN-38 is liberated from the linker during manufacturing. Once prepared and lyophilized, IMMU-132 has been stable for several years.

**Table 1:** DAR distribution and DAR_HIC_

	Percent of total			DAR_HIC_^a^
	DAR = 6	DAR = 7	DAR = 8	
Lot 1	7.0	30.5	62.5	7.56
Lot 2	7.4	27.2	65.4	7.58
Lot 3	7.1	27.7	65.3	7.59
Lot 4	6.9	24.1	69.0	7.62
Lot 5	7.0	23.0	70.0	7.63
Mean	7.1	26.5	66.4	7.60
Std. Dev.	0.2	3.0	3.0	0.03

a Average DAR calculated as the sum of fractional amount of each species determined by HIC.

**Table 2:** DAR distribution and average DAR determined by different methods

DAR	LC-MS^a^	HIC^b^
8	69.2	69.0
7	26.1	24.1
6	4.3	6.9
5	0.4	ND
Ave	7.64	7.62

a DAR species percentages and average were determined by probability mass function applied to the LC-MS results.

b DAR species percentages and average determined by HIC-HPLC method.

### Effect of DAR on pharmacokinetics and anti-tumor efficacy in mice

**Figure 3:**
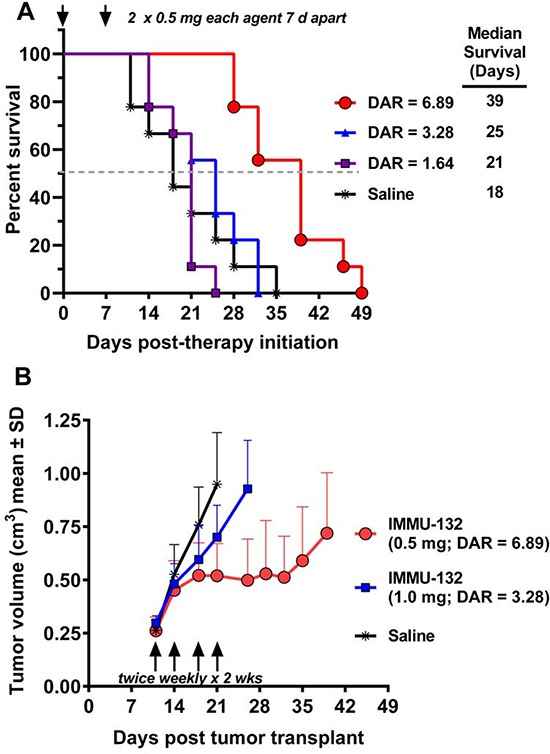
Therapeutic efficacy of IMMU-132 with different DARs NCI-N87 human gastric carcinoma xenografts (subcutaneous) were established as described in Materials and Methods. **A.** Four groups of mice (*N* = 9) were injected IV with 2 × 0.5 mg (arrows) of IMMU-132 conjugates prepared with a DAR = 6.89, 3.28, or 1.64. Control animals received saline. Therapy began 7 days after tumor cells were administered (size was 0.248 ± 0.047cm^3^). Survival curves were generated based on the time to progression to ≥ 1.0 cm^3^, and were analyzed by log-rank test (significance at *P* ≤ 0.05). **B.** NCI-N87 tumor-bearing mice (*N* = 7–9; starting size = 0.271 ± 0.053cm^3^) were treated with either 0.5 mg IMMU-132 (DAR = 6.89) or 1.0 mg DAR = 3.28 twice weekly for two weeks (arrows). Mice were euthanized and deemed to have succumbed to disease once tumors grew to > 1.0 cm^3^. Profiles of individual tumor growth were obtained through linear-curve modeling. Statistical analysis of tumor growth was based on area under the curve (AUC) performed up to the time that the first animal within a group was euthanized due to disease progression. An *f*-test was employed to determine equality of variance between groups prior to statistical analysis of growth curves. A two-tailed *t*-test was used to assess statistical significance between the various treatment groups and controls, except for the saline control, where a one-tailed *t*-test was used (significance at *P* ≤ 0.05).

Mice bearing Trop-2^+^ human gastric carcinoma xenografts (NCI-N87) were given 2 treatments 7 days apart, each with equal protein (0.5 mg) doses of IMMU-132 having DARs of 6.89, 3.28, or 1.64 (Fig 3A). Animals treated with the ADCs having a DAR of 6.89 had a significantly improved median survival time (MST) compared to mice given ADCs with either 3.38 or 1.64 DARs (MST = 39 days *vs*. 25 and 21 days, respectively; *P* < 0.0014). There was no difference between groups treated with the 3.28 or 1.64 DAR conjugates and the saline control group.

To further elucidate the importance of a higher DAR, mice bearing NCI-N87 gastric tumors were administered 0.5 mg IMMU-132 with a DAR of 6.89:1 twice weekly for two weeks (Fig 3B). Another group received twice the protein (1 mg) dose of an IMMU-132 conjugate with a DAR of 3.28. Although both groups received the same total amount of SN-38 (36 μg) with each dosing scheme, those treated with the 6.89 DAR conjugate inhibited tumor growth significantly more than tumor-bearing animals treated with the 3.28 DAR conjugate (*P* = 0.0227; AUC). Additionally, treatment with the 1.64 DAR conjugate was not significantly different than the untreated controls. Collectively, these studies indicate that a lower DAR reduces efficacy.

An examination of the pharmacokinetic behavior of the fully substituted conjugate was performed in non-tumor-bearing mice given 0.2 mg of IMMU-132, with comparison to unconjugated hRS7 IgG, as well as the reduced and then capped IgG with N-ethylmaleimide to evaluate whether breaking the interchain disulfides destabilizes the IgG in serum [37]. Serum was taken at 5 intervals from 0.5 to 168 h and assayed by ELISA for hRS7 IgG. No significant difference in the clearance of these 3 products was found, indicating that neither the disruption of the interchain nor the coupling of 8 CL2A-SN-38 linkers affected clearance.

### Trop-2 expression in TNBC and SN-38 sensitivity

**Figure 4:**
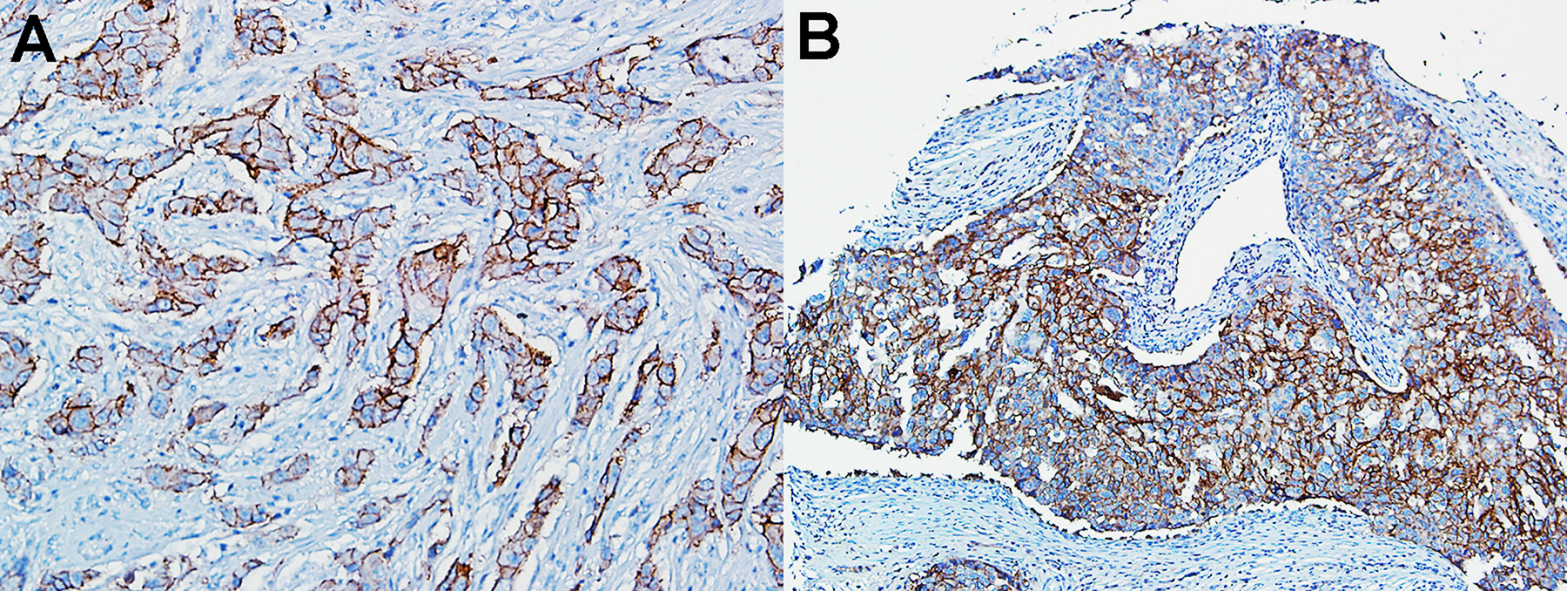
Immunohistology of TNBC patient specimens Strong (3+) Trop-2 expression in two TNBC specimens within a tumor microarray.

Trop-2 expression was determined by immunohistochemistry (IHC) in several tissue microarrays of human tumor specimens. In one microarray containing 31 TNBC specimens, as well as 15 hormone-receptor- or HER-2-positive breast cancers, positive staining occurred in over 95% of the tumors, with 3+ staining in 65% of the cases. IHC staining of two TNBC specimens with 3+ staining is shown in Fig 4.

Table 3 lists 6 human breast cancer cell lines, including four TNBC, showing their surface expression of Trop-2 and sensitivity to SN-38. Trop-2 surface expression in 5 of the 6 cell lines exceeded 90,000 copies per cell. SN-38 potency ranged from 2 to 6 nM in 5 of the 6 cell lines, with MCF-7 having the lowest sensitivity of 33 nM. *In vitro* potency for IMMU-132 is not provided, because nearly all of the SN-38 associated with IMMU-132 is released into the media during the 4-day incubation period, and therefore its potency would be similar to that of SN-38. A different strategy was required to illustrate the importance of antibody targeting as a mechanism for delivering SN-38. Antigen-positive (HCC1806) or -negative (HCC1395) TNBC cell lines were incubated at 4°C for 30 min with either IMMU-132 or a non-binding anti-CD20 SN-38 conjugate. The cells were then washed to remove unbound conjugate and incubated overnight at 37°C. Cells were fixed and permeabilized, and then stained with the fluorescent anti-phospho-histone H2A.X antibody to detect dsDNA breaks by flow cytometry [38] (Table 4). The Trop-2^+^ breast cancer cell line, HCC1806, when incubated with IMMU-132, had an increase in median fluorescence intensity (MFI) from 168 (untreated baseline) to 546, indicating the increased presence of dsDNA breaks, whereas the MFI for cells incubated with the non-binding conjugate remained at baseline levels. In contrast, MFI for the Trop-2 antigen-negative cell line, HCC1395, remained at baseline levels following treatment with either IMMU-132 or the non-binding control conjugate. Thus, the specificity of IMMU-132 over an irrelevant ADC was conclusively revealed by evidence of dsDNA breaks only in Trop-2-expressing cells incubated with the anti-Trop-2-binding conjugate.

**Table 3:** Trop-2 expression and SN-38 sensitivity in breast cancer cell lines

Cell Line	Receptor status	Trop-2 surface expression^a^	IC_50_ (nM) SN-38
SK-BR-3	HER2^+^	328,281 ± 47, 996	2
MDA-MB-468	TNBC	301,603 ± 29, 470	2
HCC38	TNBC	181,488 ± 69, 351	2
MCF-7	ER^+^	110,646 ± 17, 233	33
HCC1806	TNBC	91,403 ± 20, 817	1
MDA-MB-231	TNBC	32,380 ± 5, 460	6

a Mean ± SD number of surface Trop-2 molecules per cell from three separate assays.

**Table 4:** Specificity of IMMU-132 anti-tumor activity *in vitro* using flow cytometry with phospho-H2AX (anti-histone)-stained cells.^a^

Treatment	Median fluorescence intensity	
	HCC1806(Trop-2^+^)	HCC1395(Trop-2^−^)
Cell alone	4.25	5.54
Cell + anti-rH2AX-AF488	168	122
Cell + IMMU-132 + anti-rH2AX-AF488	546	123
Cell + hA20-SN38 + anti-rH2AX-AF488	167	123

a HCC1806 (Trop-2^+^) or HCC1395 (Trop-2^−^) were incubated at 4°C with IMMU-132 or a non-binding control conjugate (anti-CD20-SN-38) for 30 min, washed and incubated overnight at 37°C in fresh drug-free media. Cells were harvested, fixed, and permeabilized, then stained with the fluorescently-conjugated anti-histone antibody (rH2AX-AF488) for detection of double-stranded DNA breaks. The median fluorescence intensity (MFI) is given for (a) background staining of the cells alone (no anti-histone antibody), (b) the background level of dsDNA breaks for the cells that had no prior exposure to the conjugates, and (c) after exposed to IMMU-132 or hA20-SN-38 conjugates.

### *In vivo* efficacy of IMMU-132 in TNBC xenografts

**Figure 5:**
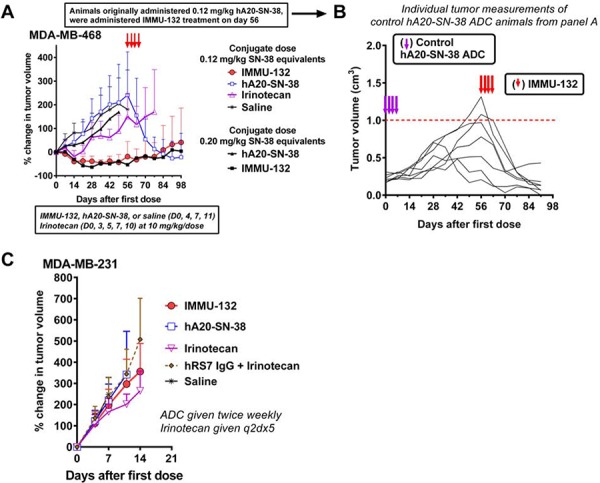
Therapeutic efficacy of IMMU-132 in TNBC xenograft models **A.** Twenty-two days after subcutaneously implantation of MDA-MB-468 tumors (at the onset of treatment, tumors averaged 0.223 ± 0.055 cm^3^), nude mice (7–8 per group) were injected with IMMU-132 or a control hA20 anti-CD20-SN-38 conjugate twice weekly for two weeks (0.12 or 0.20 mg/kg SN-38 equivalents per dose). Other animals were given irinotecan (10 mg/kg/dose; SN-38 equivalent based on mass = 5.8 mg/kg) every other day for 10 days for a total of five injections. Statistical analysis performed in the same manner as mentioned in Fig 2B. **B.** Starting on day 56 after treatment initiation, all animals in the control hA20-SN-38 group were given IMMU-132 (4 × 0.2 mg/kg SN-38 equivalents). The size of the tumors in the individual animals of this group from the onset of tumor transplantation is given. Purple arrows indicate when the hA20-SN-38 conjugate was first given, and red arrows indicate when the treatment with IMMU-132 was initiated. **C.** Mice (*N* = 12) bearing the MDA-MB-231 TNBC cell line (0.335 ± 0.078 cm^3^) were treated with IMMU-132 or the control hA20-SN-38 conjugate (0.4 mg/kg SN-38 equivalents), irinotecan (6.5 mg/kg; ∼3.8 mg/kg SN-38 equivalents), or a combination of hRS7 IgG (25 mg/kg) plus irinotecan (6.5 mg/kg).

The efficacy of IMMU-132 was assessed in nude mice bearing MDA-MB-468 TNBC tumors (Fig 5A). IMMU-132 at a dose of 0.12 or 0.20 mg/kg SN- 38-equivalents (0.15 and 0.25 mg IMMU-132/dose) induced significant tumor regression, compared to saline, irinotecan (10 mg/kg; ∼5.8 mg/kg SN-38 equivalents by weight), or a control anti-CD20 ADC, hA20-CL2A-SN-38, given at the same 2 dose levels (AUC, *P* < 0.0017). Since mice convert irinotecan to SN-38 more efficiently than humans [39] (in our studies, the conversion rate averaged ∼25%), at this irinotecan dose, ∼145 to 174 μg of SN-38 would be produced, while the administered dose of IMMU-132 contained only 9.6 μg. Nevertheless, because IMMU-132 selectively targeted SN-38 to the tumors, it was more efficacious. These results corroborate findings in other solid tumor models [2] showing that specific targeting of a small amount of SN-38 to the tumor with IMMU-132 is much more effective than a much larger dose of irinotecan, or a mixture of hRS7 IgG with an equal amount of free SN-38 [2]. The unconjugated RS7 antibody, even at repeated doses of 1 mg per animal, did not show any antitumor effects [2]. However, *in-vitro* studies with gynecological cancers expressing Trop-2 have indicated cell killing with the RS7 mAb by antibody-dependent cellular cytotoxicity [15, 40–42]. Two other unconjugated anti-Trop-2 antibodies have been reported to be active therapeutically in preclinical testing, one as a Fab fragment [43, 44].

On therapy day 56, four of the seven tumors in mice given 0.12 mg/kg of the hA20-CL2A-SN-38 control ADC had progressed to the endpoint of 1.0 cm^3^ (Fig 5B). At this time, these animals were treated with IMMU-132, electing to use the higher dose of 0.2 mg/kg in an attempt to affect the progression of these much larger tumors. Despite the substantial size of the tumors in several animals, all mice demonstrated a therapeutic response, with tumors significantly smaller five weeks later (tumor volume [TV] = 0.14 ± 0.14 cm^3^ *vs*. 0.74 ± 0.41 cm^3^, respectively; *P* = 0.0031, two-tailed *t*-test). Similarly, we chose two animals in the irinotecan-treated group with tumors that progressed to ∼0.7 cm^3^ and re-treated one with irinotecan and the other with IMMU-132 (not shown). Within 2 weeks of ending treatment, the tumor in the irinotecan-treated animal decreased 23% and then began to progress, while the animal treated with IMMU-132 had a 60% decrease in tumor size. These results demonstrate that even in tumors that continued to grow after exposure to SN-38 *via* a non-specific ADC, a significantly enhanced therapeutic response could be achieved when treated with the Trop-2-specific IMMU-132. However, specific therapeutic effects with IMMU-132 were not achieved in MDA-MB-231 (Fig 5C). As shown previously in Table 3, this cell line had the lowest Trop-2 levels, and also was the least sensitive to SN-38 as a free drug. Thus, Trop-2 expression alone will not define whether a given cell line will respond to treatment. Other factors, such as the percentage of cells expressing the antigen and their distribution in the tumor, in addition to the tumor's natural resistance to a given drug and a variety of physiological factors, will likely govern the effects of a targeted therapeutic *in vivo*.

### Mechanism of action of IMMU-132 in TNBC

**Figure 6:**
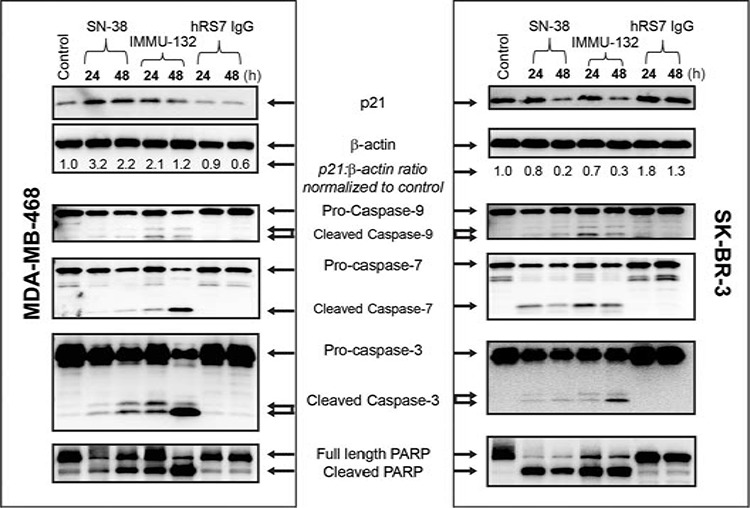
IMMU-132 mediated pro-apoptosis signaling in human breast cancer lines MDA-MB-468 or SK-BR-3 cells were exposed to 1 μM SN-38, the SN-38-equivalent of IMMU-132, or protein equivalent of hRS7 for the indicated times. Cells were harvested and Western blots performed as described in Materials and Methods. Untreated control cells (control) were maintained in growth medium alone until harvested after 48 h. Blots shown are representative of one of two separate experiments.

The apoptotic pathway utilized by IMMU-132 was examined in the TNBC cell line, MDA-MB-468, and in the HER2^+^ SK-BR-3 cell line, in order to confirm that the ADC functions on the basis of its incorporated SN-38 (Fig 6). Both SN-38 alone and IMMU-132 mediated > 2-fold up-regulation of p21^WAF1/Cip1^ within 24 h in MDA-MB-468, and by 48 h, the amount of p21^WAF1/Cip1^ in these cells began to decrease (31% and 43% with SN-38 or IMMU-132, respectively). Interestingly, in the HER2^+^ SK-BR-3 tumor line, neither SN-38 nor IMMU-132 mediated the up-regulation of p21^WAF1/Cip1^ above constitutive levels in the first 24 h, but as seen in MDA-MB-468 cells after 48-h exposure to SN-38 or IMMU-132, the amount of p21^WAF1/Cip1^ decreased > 57%. Both SN-38 and IMMU-132 resulted in cleavage of pro-caspase-3 into its active fragments within 24 h, but with the greater degree of active fragments observed after exposure for 48 h. Of note, in both cell lines, IMMU-132 mediated a greater degree of pro-caspase-3 cleavage, with the highest level observed after 48 h when compared to cells exposed to SN-38. Finally, SN-38 and IMMU-132 both mediated poly ADP ribose polymerase (PARP) cleavage, starting at 24 h, with near complete cleavage after 48 h. Taken together, these results confirm that IMMU-132 has a mechanism of action similar to that of free SN-38 when administered *in vitro*.

### Delivery of SN-38 by IMMU-132 vs. irinotecan in a human tumor xenograft model

Constitutive products derived from irinotecan or IMMU-132 were determined in the serum and tumors of mice implanted subcutaneously with a human pancreatic cancer xenograft (Capan-1) and then administered irinotecan (773 μg; SN-38 equivalents = 448 μg) and IMMU-132 (1.0 mg; SN-38 equivalents = 16 μg).

**Figure 7:**
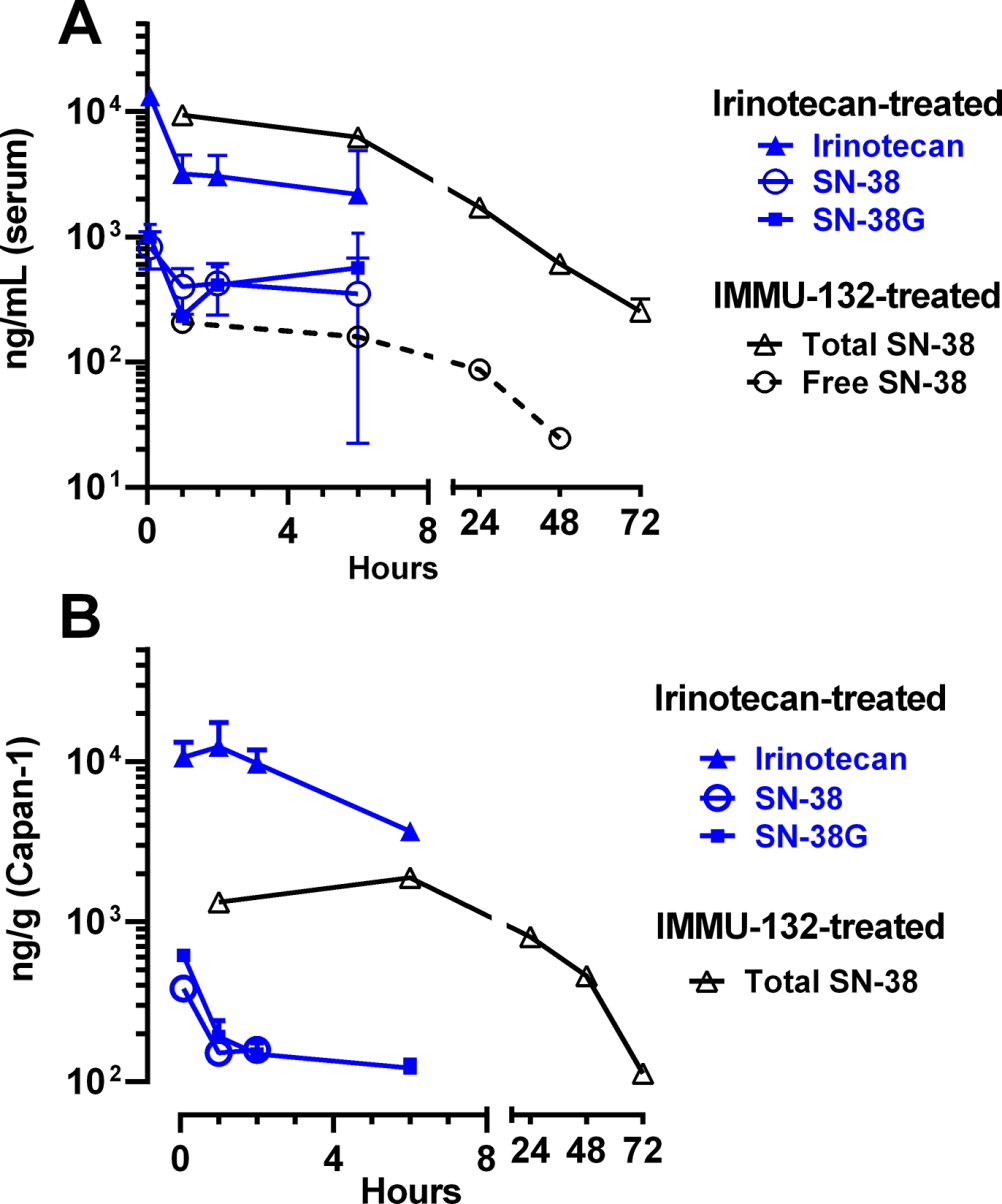
Determination of SN-38 and associated products in serum and Capan-1 tumors taken from animals given irinotecan or IMMU-132 Animals were given irinotecan (773 μg) or IMMU-132 (1.0 mg) and then at 5 intervals, 3 animals from each group were euthanized with serum panel **(A)** and tumor panel **(B)** extracted for the products of interest. Data are only shown for sampling intervals where detectable product was measured (e.g., animals given irinotecan did not have any detectable products at the 24-h sampling interval).

Irinotecan cleared very rapidly from serum, with conversion to SN-38 and glucuronidated SN-38 (SN-38G) seen within 5 min (Fig 7A). None of the products was detected at 24 h. The AUCs over a 6-h period were 21.0, 2.5, and 2.8 h.μg/mL for irinotecan, SN-38, and SN-38G, respectively (SN-38 conversion in mice = [2.5 + 2.8)/21 = 25.2%]). Animals given IMMU-132 had much lower concentrations of free SN-38 in the serum, but it was detected through 48 h. Free SN-38G was detected only at 1 and 6 h, and was 3- to 7-times lower than free SN-38.

In the Capan-1 tumors excised from irinotecan-treated animals, irinotecan levels were high over 6 h, but undetected at 24 h (AUC_5min-6 h_ = 48.4 h.μg/g). SN-38 was much lower and detected only through 2 h (i.e., AUC_5min-2 h_ = 0.4 h.μg/g), with SN-38G values almost 3-fold higher (AUC = 1.1 h.μg/g) (Fig 7B). Tumors taken from animals given IMMU-132 did not have any detectable free SN-38 or SN-38G, but instead, all SN-38 in the tumor was bound to IMMU-132. Importantly, since no SN-38G was detected in the tumors, this suggests SN-38 bound to IMMU-132 was not glucuronidated. The AUC for SN-38 bound to IMMU-132 in these tumors was 54.3 h.μg/g, which is 136-fold higher than the amount of SN-38 in the tumors of animals treated with irinotecan over the 2-h period that SN-38 could be detected, even though mice given irinotecan received 28-fold more SN-38 equivalents than administered with IMMU-132 (i.e., 448 *vs*. 16 μg SN-38 equivalents, respectively).

## DISCUSSION

We describe a new ADC targeting Trop-2, and early clinical results suggest it is well tolerated and effective in patients with TNBC, as well as other Trop-2^+^ cancers [25]. Due to its distinct properties, IMMU-132 represents a second-generation ADC. Typically, ADCs require 4 broad attributes to be optimally effective [33, 34, 45, 46]: (i) selective targeting/activity; (ii) binding, affinity, internalization, and immunogenicity of the antibody used in the ADC; (iii) the drug, its potency, metabolism and pharmacological disposition, and (iv) how the drug is bound to the antibody. Target selectivity is the most common requirement for all ADCs, since this will play a major role in defining the therapeutic index (ratio of toxicity to tumor *vs*. normal cells); the choice of the binding epitope of the target antigen and the binding kinetics are general challenges to every system. Trop-2 appears to have both a high prevalence on a number of epithelial cancers, but it is also expressed by several normal tissues [12, 13, 47], which could have impacted specificity. However, expression in normal tissues appears to be lower than in cancers [15], and Trop-2 appears to be shielded in some normal tissues by their architecture that limits accessibility to an antibody, whereas in cancer, these tissue barriers are compromised by the invading tumor. Evidence of this was apparent from initial toxicological studies in monkeys, where despite escalating IMMU-132 doses to levels leading to irinotecan-like neutropenia and diarrhea, histopathological damage to Trop-2-expressing normal tissues did not occur [2]. These results appear to have been confirmed clinically, where no specific organ toxicity has been noted in patients to-date, except for the known toxicities of the parental compound, irinotecan [25], which are more manageable with IMMU-132.

A generally accepted and important criterion for ADC therapy is that the antibody should internalize, delivering its chemotherapeutic inside the cell, where it is usually metabolized in lysosomes. Despite IMMU-132′s internalization, we believe that the linker in this ADC, which affords local release of SN-38, is likely another feature that sets this platform technology apart from those using an ultratoxic drug, because the released free drug can induce a bystander effect on neighboring cancer cells. Indeed, having an ultratoxic agent linked stably to the IgG is the only configuration that would preserve a useful therapeutic window for those types of compounds. However, using a more moderately-toxic drug does not give the latitude to use a linker that would release the drug too early once in the circulation. Our group explored linkers that released SN-38 from the conjugate with different half-lives in serum, ranging from ∼10 h to a highly stable linker, but it was the linker with the intermediate stability that provided the best therapeutic response in mouse-human tumor xenograft models [30, 31]. Since this initial work, we showed that a highly stable linkage of SN-38 was significantly less effective than the CL2A linker that has a more intermediate stability in serum [35].

Another current tenet of ADC design is to use an ultra-cytotoxic drug to compensate for low levels of antibody accretion in tumors, typically 0.003 to 0.08% of the injected dose per gram [36]. The current generation of ultratoxic-drug conjugates has found a drug:antibody substitution of ≤ 4:1 to be optimal, since higher ratios adversely affected their pharmacokinetics and diminished the therapeutic index by collateral toxicities [48]. In this second-generation ADC technology, we elected to use an IgG-coupling method that site-specifically links the drug to the interchain disulfides through mild reduction of the IgG, which exposes 8 binding sites. With the CL2A-SN-38 linker, we achieved a DAR of 7.6:1, with LC-MS data showing each of the 8 coupling sites bears the CL2A linker, but apparently some SN-38 is lost during the manufacturing procedure. Nevertheless, 95% of the CL2A linker has 7–8 SN-38 molecules. We found subsequently that coupling to these sites does not destabilize the antibody, which is consistent with the findings by Rispens et al. [49], who showed the affinity of the interaction between C_H_3 domains of the heavy chains of human IgG1 is between 10^−13^ and 10^−14^ M. Conjugates prepared with these sites substituted at higher levels also did not compromise antibody binding, nor did it affect pharmacokinetic properties. Indeed, we demonstrated that conjugates prepared at the maximum substitution level had the best therapeutic response in mouse-human tumor xenograft models.

One of the more notable features of IMMU-132 from a tolerability perspective is that the SN-38 bound to IgG is not glucuronidated, which is a critical step in the detoxification of irinotecan. With irinotecan therapy, most of the SN-38 generated is readily converted in the liver to the inactive SN-38G form. Estimates of the AUC for SN-38G show it is often 4.5- to 32-times higher than SN-38 [50, 51]. SN-38G's secretion into the bile and subsequent deconjugation by beta-glucuronidase produced by the intestinal flora is strongly implicated in the enterohepatic recirculation of SN-38 and the delayed severe diarrhea observed with irinotecan [52]. After IMMU-132 administration, concentrations of SN-38G were very low in our animal and clinical studies (e.g., in the serum of patients given IMMU-132, only 20–40% of the free SN-38 levels are in the form of SN-38G), providing strong evidence that SN-38 bound to IgG is largely protected from glucuronidation, even though the 10-hydroxy position of the SN-38 is available. We speculate that low levels of SN-38G generated by IMMU-132 contributes to the lower incidence and intensity of diarrhea in patients receiving this ADC compared to irinotecan therapy.

Preventing glucuronidation of the SN-38 bound to the antibody may also contribute to improved therapeutic effects for SN-38 delivered to the tumor. Extracts of tumors from animals given irinotecan found high levels of irinotecan, with 10-fold lower concentrations of SN-38 and SN-38G. In contrast, the only SN-38 found in the tumors of animals given IMMU-132 was SN-38 bound to the IgG. We hypothesize that the conjugate retained in the tumor will eventually be internalized, thereby releasing its SN-38 payload, or SN-38 could be released outside the tumor cell; however, it would be released in its fully active form, with a lower likelihood of being converted to SN-38G, which occurs primarily in the liver. It is also important to emphasize that by coupling the linker to the 20-hydroxy position of SN-38, the SN-38 is maintained in the active lactone form [32]. Collectively, these results suggest that IMMU-132 is able to deliver and concentrate SN-38 to Trop-2^+^ tumors in a selective manner compared to SN-38 derived from non-targeted irinotecan, with the SN-38 delivered by IMMU-132 likely being released in the tumor in the fully active, non-glucuronidated, lactone form.

Irinotecan is not conventionally used to treat breast cancer patients. However, the experiments shown here with TNBC cell lines indicate that concentrating higher amounts of SN-38 into the tumor enhances its activity. In both the MDA-MB-468 TNBC and HER2^+^ SK-BR-3 tumor lines, IMMU-132 mediated the activation of the intrinsic apoptotic pathway, with cleavage of pro-caspases into their active fragments and PARP cleavage. The demonstration of double-stranded DNA breaks of cancer cells treated with IMMU-132 (compared to an irrelevant SN-38 ADC) confirms the selective delivery of SN-38 into the target cells. Most importantly, these laboratory findings are corroborated by our translation of this ADC therapy to patients with heavily-pretreated, metastatic TNBC, where durable objective responses have been observed [25]. It also appears that IMMU-132 is active in patients with other cancers and who have failed a prior therapy regimen containing a topoisomerase I inhibitor [24].

In conclusion, our results suggest that Trop-2 is a clinically-relevant and novel target in many solid tumors, particularly TNBC as well as lung cancers [25]. IMMU-132 appears to be a novel, second-generation ADC by using a moderately-toxic drug, SN-38, conjugated at a high ratio of drug to an antibody via a moderately-stable linker. This ADC appears to be more tolerable than when the parental drug, irinotecan, is administered [24], and thus challenges certain tenets of current ADC practice.

## MATERIALS AND METHODS

### Properties of IMMU-132

The design rationale and synthesis of SN-38 derivatives for antibody conjugation have been described previously [2, 30]. Briefly, CL2A-SN-38, with the attachment of the linker at the drug's 20-hydroxy position to avoid or minimize the lactone ring opening to the inactive form, involved first forming the carbonate linkage to the drug using an azido-PEG-lysyl-*p*-amidobenzyl alcohol linker, followed by cycloaddition to a maleimide-incorporated acetylene derivative to attach the antibody-conjugating group. In the CL2A linker, the incorporation of a short polyethylene glycol (PEG) moiety enabled aqueous solubility of highly insoluble SN-38.

### DAR determination by HIC

Clinical lots of IMMU-132 were analyzed by hydrophobic interaction chromatography (HIC) using a butyl-NPR HPLC column (Tosoh Bioscience, King of Prussia, PA). IMMU-132 injections (100 μg) were resolved with a 15-min linear gradient of 2.25–1.5 M NaCl in 25 mM sodium phosphate, pH 7.4, run at 1 mL/min and room temperature.

### DAR determination by LC-MS

Because the interchain disulfides are reduced and the resulting sulfhydryl groups are used for drug conjugation (or blocked), the heavy and light chains resolved during LC-MS analysis without addition of reducing agents, and were analyzed independently. Different lots of IMMU-132 were injected on an Agilent 1200 series HPLC using an Aeris Widepore C4 reverse-phase HPLC column (3.6 μM, 50 × 2.1 mm) and resolved by reverse phase HPLC with a 14-min linear gradient of 30 – 80% acetonitrile in 0.1% formic acid. Electrospray ionization time of flight (ESI-TOF) mass spectrometry was accomplished with an in-line Agilent 6210 ESI-TOF mass spectrometer with Vcap, fragmentor and skimmer set to 5000V, 300V and 80V, respectively. The entire RP-HPLC peak representing all kappa or heavy chain species were used to generate deconvoluted mass spectra.

### Cell lines

All human cancer cell lines used in this study were purchased from the American Type Culture Collection (Manassas, VA), except where noted. Each cell line was maintained according to the recommendations of ATCC and routinely tested for mycoplasma using MycoAlert^®^ Mycoplasma Detection Kit (Lonza; Rockland, ME) and all were authenticated by short tandem repeat (STR) assay by the ATCC.

### Trop-2 surface expression on various human breast carcinoma cell lines

Expression of Trop-2 on the cell surface is based on flow cytometry. Briefly, cells were harvested with Accutase Cell Detachment Solution (Becton Dickinson (BD); Franklin Lakes, NJ; Cat. No. 561527) and assayed for Trop-2 expression using QuantiBRITE PE beads (BD Cat. No. 340495) and a PE-conjugated anti-Trop-2 antibody (eBiosciences, Cat. No. 12-6024) following the manufacturer's instructions. Data were acquired on a FACSCalibur Flow Cytometer (BD) with CellQuest Pro software, with analysis using Flowjo software (Tree Star; Ashland OR).

### IHC of Trop-2 in tumor microarrays

A TNBC microarray was purchased from US Biomax (Rockville, MD; product BR487). Antigen retrieval was done by incubating the slides in a Tris/EDTA buffer (DaKo Target Retrieval Solution, pH 9.0; Dako, Denmark), at 95°C in a NxGen Decloaking Chamber (Biocare Medical; Concord, CA) for 30 minutes. Trop-2 was detected with a goat polyclonal anti-human Trop-2 antibody at 10 μg/mL (R&D Systems, Minneapolis, MN) and stained with VECTASTAIN^®^ ABC Kit (Vector Laboratories, Inc.; Burlingame, CA). Normal goat antibody was used as the negative control (R&D Systems, Minneapolis, MN). Tissues were counterstained with hematoxylin. A formalin-fixed, paraffin-embedded section from a xenograft of the BxPC3 human pancreatic cancer cell line served as a positive control. Scoring was based on the intensity of the stain in > 10% of the tumor cells within the specimen, including negative, 1+ (weak), 2+ (moderate), and 3+ (strong).

### *In vitro* cytotoxicity testing

Sensitivity to SN-38 was determined using the 3-(4, 5-dimethylthiazol-2-yl)-5-(3-carboxymethoxyphenyl)-2-(4-sulfophenyl)-2H-tetrazolium dye reduction assay (MTS dye reduction assay; Promega, Madison, WI). Briefly, cells were plated into 96-well clear, flat-bottomed plates as described above. SN-38 dissolved in DMSO was diluted with media to a final concentration of 0.004 to 250 nM. Plates were incubated in humidified chamber for 96 h at 37°C/5% CO_2_, after which the MTS dye was added and placed back into the incubator until untreated control cells had an absorbance greater than 1.0. Growth inhibition was measured as a percent of growth relative to untreated cells. Dose-response curves were generated from the mean of triplicate determinations, and IC_50_-values were calculated using Prism GraphPad Software.

### *In vitro* specificity testing by flow cytometry with rH2AX-stained cells

For drug activity testing, HCC1806 and HCC1395 TNBC cell lines cells were seeded in 6-well plates at 5 × 10^5^ cells/well and held at 37°C overnight. After cooling the cells for 10 min on ice, the cells were incubated with either IMMU-132 or hA20 anti-CD20-SN38 at ∼20 μg/ml (equal SN38 /well for both agents) for 30 minutes on ice, washed three times with fresh media, and then returned to 37°C overnight. Cells were trypsinized briefly, pelleted by centrifugation, fixed in 4% formalin for 15 min, then washed and permeabilized in 0.15% Triton-X100 in PBS for another 15 min. After washing twice with 1% bovine serum albumin-PBS, cells were incubated with mouse anti-rH2AX-AF488 (EMD Millipore Corporation, Temecula, CA) for 45 minutes at 4°C. The signal intensity of rH2AX was measured by flow cytometry using a BD FACSCalibur (BD Biosciences, San Jose, CA).

### *In vivo* therapeutic studies in xenograft models

NCr female athymic nude (*nu*/*nu*) mice, 4–8 weeks old, were purchased from Taconic Farms (Germantown, NY). Capan-1 xenografts were established by making a tumor suspension from stock tumors and mixing with cells harvested from tissue culture. A 0.3mL injection, containing 20% w/v tumor suspension plus 1 × 10^7^ cells, was injected subcutaneously into the flank. NCI-N87 and MDA-MB-468 tumors were established by harvesting cells from tissue culture and making a final cell suspension by mixing 1:1 with matrigel (BD Bioscience; San Jose, CA) such that each mouse received a total of 1 × 10^7^ cells subcutaneously in the right flank. Tumor volume (TV) was determined by measurements in two dimensions using calipers, with volumes defined as: *L* × *w*^2^/2, where *L* is the longest dimension of the tumor and *w* the shortest. Mice were randomized into treatment groups and therapy begun when tumor volumes were approximately 0.25 cm^3^. Treatment regimens, dosages, and number of animals in each experiment are described in the Results and in the Figure Legends. The lyophilized IMMU-132 and control ADC (hA20-CL2A-SN-38) were reconstituted and diluted as required in sterile saline. SN-38 equivalents in a dose of 250 μg ADC to a 20-gram mouse (12.5 mg/kg) is equal to 0.2 mg SN-38/kg. For irinotecan (irinotecan-HCl injection; AREVA Pharmaceuticals, Inc., Elizabethtown, KY), 10 mg irinotecan/kg converts to 5.8 mg SN-38/kg based on mass.

Mice were euthanized and deemed to have succumbed to disease once tumors grew to greater than 1.0 cm^3^ in size. Statistical analysis of tumor growth was based on area under the curve (AUC). Profiles of individual tumor growth were obtained through linear-curve modeling. An *f*-test was employed to determine equality of variance between groups prior to statistical analysis of growth curves. A two-tailed *t*-test was used to assess statistical significance between the various treatment groups and controls, except for the saline control, where a one-tailed *t*-test was used (significance at *P* ≤ 0.05). Statistical comparisons of AUC were performed only up to the time that the first animal within a group was euthanized due to disease progression. Survival was analyzed by log-rank test on survival curves generated for each treatment (significance at *P* ≤ 0.05).

### Immunoblotting

Cells (2 × 10^6^) were plated in 6-well plates overnight. The following day they were treated with either SN-38 or IMMU-132 at an SN-38 concentration equivalent of 0.4 μg/mL (1 μM) for 24 and 48 h. Parental hRS7 was used as a control for the ADC. Cells were lysed in buffer containing 10 mM Tris, pH 7.4, 150 mM NaCl, protease inhibitors and phosphatase inhibitors (2 mM Na_2_PO_4_, 10 mM NaF). A total of 20 μg protein was resolved in a 4–20% SDS polyacrylamide gel, transferred onto a nitrocellulose membrane and blocked by 5% non-fat milk in 1× TBS-T (Tris-buffered saline, 0.1% Tween-20) for 1 h at room temperature. Membranes were probed overnight at 4°C with primary antibodies followed by 1-h incubation with anti-rabbit secondary antibody (1:2500) at room temperature. Signal detection was done using a chemiluminescence kit (Supersignal West Dura, Thermo Scientific; Rockford, IL) with the membranes visualized on a Kodak Image Station 40000R. Primary antibodies p21^Waf1/Cip1^ (Cat. No. 2947), Caspase-3 (Cat. No. 9665), Caspase-7 (Cat. No. 9492), Caspase-9 (Cat. No. 9502), PARP (Cat. No. 9542), β-actin (Cat. No. 4967), and goat anti-rabbit-HRP secondary antibody (Cat. No. 7074) were obtained from Cell Signal Technology (Danvers, MA).

### Quantification of SN-38 in mice with human tumor xenografts

Groups of Capan-1-tumor bearing animals were given a single intravenous injection of irinotecan (773 μg; SN-38 equivalents = 448 μg) or IMMU-132 (1.0 mg; SN-38 equivalents = 16 μg). Animals were euthanized at 5 min, 1, 2, 6, and 24 h for the irinotecan group, while mice given IMMU-132 were analyzed at 1, 6, 24, 48, and 72 h (*N* = 3 per interval per group). In irinotecan-treated animals, water homogenates of the tumor (1 part tumor + 10 parts water) or serum diluted 1:1 in water were extracted and analyzed by reversed-phase HPLC for irinotecan, SN-38, and SN-38 glucuronide (SN-38G). Each sample (150 μL diluted serum or tissue homogenate that was not clarified by centrifugation prior to extraction) was first spiked with an internal standard (9 μL of 3.33 μg/mL of 10-hydroxycamptothecin), and then mixed with 150 μL of an extraction/protein-precipitation reagent (1:1:1 methanol, ethylene glycol and 1 M ZnSO_4_ in DI water) as described previously by Hirose et al. [53]. After vortexing, the sample was centrifuged for 10 min (8500 rpm), and an aliquot (5–20 μL) of the supernatant was analyzed using an EMD Millipore Chromolith High Resolution RP-18e 100–4.6 column. The column was eluted at 1 mL/min using a gradient (Buffer A: 50 mM KH_2_PO_4_ and 4 mM sodium-1-decanesulfonate in DI water, pH 3.5 and Buffer B: 60% Buffer A plus 40% acetonitrile) starting with 85:15 A/B running to 100% B over 15 min. The HPLC eluent was monitored with a fluorescence detector, using 373 nm excitation and 540 nm emission wavelengths. Additional details are provided by Sharkey et al. [54].

While spiking studies found the extraction method was suitable for isolating SN-38 and SN-38G (> 90% recovery), when IMMU-132 was extracted, the full content of SN-38 contained in the sample was not recovered, because the SN-38 bound to the hRS7 IgG was precipitated with the other proteins. Therefore, an acid-hydrolysis step was introduced prior to extraction, where 150 μL of 6M HCl was added to 150 μL of the samples, each containing the internal standard. The sample was incubated at 50°C overnight, and then neutralized with 111 μL 6 M NaOH, which was then mixed with 300 μL of the extraction/protein-precipitation reagent. The sample was then centrifuged and analyzed by HPLC as described above. Qualification assessments found that > 90% of the expected SN-38 bound to the IgG was released and identified by this procedure. Thus, the analysis of the non-hydrolyzed sample detected free SN-38 in the samples taken from IMMU-132-treated animals, while the acid-hydrolyzed samples detected the IMMU-132-associated SN-38 in the sample along with the free SN-38. Since studies found < 5% of the SN-38 in the IMMU-132-containing samples was free SN-38, this value essentially represents IMMU-132 SN-38. Samples taken from animals given irinotecan were analyzed only without acid-hydrolysis.

Quantitation of the drug products was based on standard curves prepared from known concentrations of each drug product of interest (i.e., SN-38, SN-38G, and irinotecan) prepared in duplicate, ranging from 10 to 10, 000 ng/mL, and examining the log of the ratio of the area under the curve (AUC) for the product of interest divided by the AUC for the internal standard plotted against the log of the product of interest (acceptance, correlation coefficient > 99%). Samples of serum and tumor from each animal were assayed at each of the times indicated, with the average concentrations plotted over time. Based on the dilution of the samples, the minimum sensitivity of detection was 20 ng/mL for serum and 110 ng/mL in tissue homogenates. AUC values were determined for the specific interval where drug products were detected using Prism (GraphPad software, version 6.0).

### Statistics

Statistical analyses were performed using GraphPad Prism version 5.00 for Windows, GraphPad Software, La Jolla, California, USA, or Microsoft Excel. The specific testing performed is identified with each study.

### Study approval

All animal studies were approved by Rutgers School of Biomedical and Health Sciences Institutional Animal Care and Use Committee.
