# Correction: Trop-2 is a novel target for solid cancer therapy with sacituzumab govitecan (IMMU-132), an antibody-drug conjugate (ADC)*

**DOI:** 10.18632/oncotarget.27512

**Published:** 2020-03-10

**Authors:** David M. Goldenberg, Thomas M. Cardillo, Serengulam V. Govindan, Edmund A. Rossi, Robert M. Sharkey

**Affiliations:** ^1^Immunomedics, Inc., Morris Plains, NJ, USA; ^*^Presented in part as a lecture by DMG, “*Challenging the Dogmas: Clinical Efficacy of SN-38-conjugated Antibodies in Solid Tumors*,” at the AACR-NCI-EORTC International Conference on Molecular Targets and Cancer Therapy, Barcelona, Spain, November 20, 2014.

**This article has been corrected:** Due to an entry error found in one of the triplicate data for the 48-h time-point of stability in human serum *in vitro*, the half-life for drug release has been corrected to 18.82 h from the stated 23.98 h in Figure 1C. The corrected Figure 1C is shown below. The authors declare that these corrections do not change the results or conclusions of this paper.

**Figure 1:**
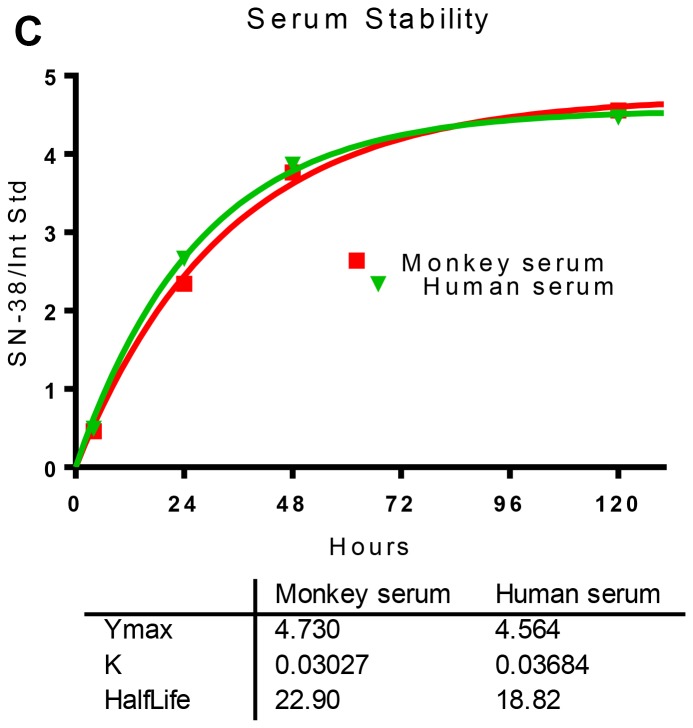
Structures of irinotecan, SN-38, and CL2A-SN-38. (**C**) *In vitro* serum stability of IMMU-132 in monkey or human serum.

Original article: Oncotarget. 2015; 6:22496–22512. 22496-22512. https://doi.org/10.18632/oncotarget.4318

